# Expression Patterns of MOTS-c in Adrenal Tumors: Results from a Preliminary Study

**DOI:** 10.3390/ijms25168721

**Published:** 2024-08-09

**Authors:** Kacper Kamiński, Małgorzata Blatkiewicz, Marta Szyszka, Anna Olechnowicz, Hanna Komarowska, Anna Klimont, Tomasz Wierzbicki, Marek Karczewski, Marek Ruchała, Marcin Rucinski

**Affiliations:** 1 Department of Histology and Embryology, Poznan University of Medical Sciences, 60-781 Poznan, Poland; k.kaminski97@gmail.com (K.K.); mblatkiewicz@ump.edu.pl (M.B.); mszyszka@ump.edu.pl (M.S.); annaolechnowicz1996@gmail.com (A.O.); 2 Doctoral School, Poznan University of Medical Sciences, 60-812 Poznan, Poland; 3 Department of Endocrinology, Metabolism and Internal Medicine, Poznan University of Medical Sciences, 60-356 Poznan, Poland; hkomar@ump.edu.pl (H.K.); anna.klimont@usk.poznan.pl (A.K.); mruchala@ump.edu.pl (M.R.); 4 Department of General, Endocrinological and Gastroenterological Surgery, Poznan University of Medical Sciences, 60-355 Poznan, Poland; tomasz.wierzbicki@ump.edu.pl; 5 Department of General and Transplantation Surgery, Poznan University of Medical Sciences, 60-356 Poznan, Poland; mkar@ump.edu.pl

**Keywords:** MOTS-c, adrenal tumors, mitochondria homeostasis

## Abstract

Adrenal tumors, such as adrenocortical carcinoma (ACC), adrenocortical adenoma (ACA), and pheochromocytoma (PCC) are complex diseases with unclear causes and treatments. Mitochondria and mitochondrial-derived peptides (MDPs) are crucial for cancer cell survival. The primary aim of this study was to analyze samples from different adrenal diseases, adrenocortical carcinoma, adrenocortical adenoma, and pheochromocytoma, and compare them with normal adrenal tissue to determine whether the expression levels of the mitochondrial open reading frame of the 12S rRNA type-c (MOTS-c) gene and protein vary between different types of adrenal tumors compared to healthy controls using qPCR, ELISA, and IHC methods. Results showed decreased *MOTS-c* mRNA expression in all adrenal tumors compared to controls, while serum MOTS-c protein levels increased in ACA and PCC but not in ACC. The local distribution of MOTS-c protein in adrenal tissue was reduced in all tumors. Notably, MOTS-c protein expression declined with ACC progression (stages III and IV) but was unrelated to patient age or sex. Tumor size and testosterone levels positively correlated with *MOTS-c* mRNA but negatively with serum MOTS-c protein. Additionally, serum MOTS-c protein correlated positively with glucose, total cholesterol, HDL, LDL, and SHGB levels. These findings suggest disrupted expression of *MOTS-c* in the spectrum of adrenal diseases, which might be caused by mechanisms involving increased mitochondrial dysfunction and structural changes in the tissue associated with disease progression. This study provides a detailed examination of MOTS-c mRNA and protein in adrenal tumors, indicating the potential role of MDPs in tumor biology and progression.

## 1. Introduction

Adrenal tumors are commonly found as incidentalomas in patients undergoing imaging for reasons other than suspected adrenal disease, and are mostly benign and hormonally inactive [1,2]. However, they can also belong to a rare group of more aggressive tumors, including adrenocortical carcinoma (ACC), pheochromocytoma (PCC), hormone-producing adrenocortical adenoma (ACA), and metastasis [3]. ACC is a rare endocrine malignancy with a poor prognosis [4]. The median age of ACC diagnosis in adults is 55 years with occurrence more common in females than in males [5,6,7]. According to current guidelines, ACC can be present in three clinical forms. The first form, the most common (40–60% of cases), manifests as symptoms of hormonal excess. The second form (approximately 30% of cases) is characterized by non-specific symptoms such as abdominal pain, bloating, or general symptoms of malignancy. However, owing the lack of clinical symptoms, a large percentage of patients (20–30%) are diagnosed on imaging studies performed for other reasons [8,9,10]. A growing body of evidence suggests that genetic factors may predispose to ACC development, such as overexpression of insulin-like growth factor 2 (*IGF2*), mutations in *TP53*, catenin beta-1 (*CTNNB1*), and the Wnt/β-catenin signaling pathway, particularly zinc and ring finger 3 (*ZNRF3*) in adults [11,12,13,14]. In addition, many ACC-related deaths are associated with metastasis, where secondary tumors have a higher mutation rate and tumor heterogeneity than primary tumors [15].

ACAs represent the majority of all adrenal tumors and are usually diagnosed predominantly in women, mainly over the age of 40 years; however, their incidence increases with age [6,7,16]. ACAs are predominantly benign and hormonally inactive, but hormonally active ACAs may produce excess androgens. Untreated excess hormone production can lead to hyperaldosteronism, Conn’s syndrome, or hypercortisolism, which are responsible for Cushing’s syndrome [17]. Untreated adrenal hormone excess is associated with increased cardiovascular risk and mortality; therefore, it is important to determine whether the adrenal mass is hormonally active [18]. 

Another type of adrenal tumor, PCCs, originates from the adrenal medulla and occurs with an incidence of 2 per 1 million adults at a mean age of 42 years, and is more common in women than in men [6,19,20,21]. PCC often carries germline or somatic gene mutations, and its symptoms are related to catecholamine overproduction or to a mass effect [21,22]. Patients with a family history are younger at diagnosis and more likely to have bilateral tumors than those with sporadic disease [19,23]. PCC is often associated with genetic syndromes such as neurofibromatosis type 1 (NF1), multiple endocrine neoplasia type 2 (MEN 2), and von Hippel–Lindau syndrome (VHL) [22]. While malignant ACC and benign ACA originate from the adrenal cortex, PCC originates from chromaffin cells in the adrenal medulla, suggesting a different molecular mechanism of tumorigenesis.

Cancer development and progression are associated with increased oxidative stress due to dysfunction of damaged mitochondria, which play an essential role in cellular homeostasis by regulating energy production and apoptosis [24,25]. In addition, mitochondria, which are abundant in the adrenal glands, especially in the adrenal cortex, are essential for steroid hormone synthesis [26]. The discovery of MDPs opens a new perspective on mitochondria function in human cells. Besides energy production, mitochondria are involved in cell death, cellular stress responses, and information transfer [27]. Thus, impaired function can lead to various pathologies and diseases, such as neurodegenerative diseases or metabolic imbalances [28]. It has been confirmed that the pathogenesis of ACC is characterized by abnormal mitochondrial metabolism and may contribute to the progression of ACC [29]. Moreover, mitochondrial morphology in adrenal adenomas adapts to the enzyme activity and steroid biosynthetic capacity of the tumor, with different structures indicating different functions [30]. Furthermore, mutations in genes related to mitochondrial function are thought to play important roles in cancer development and metastasis [31,32]. Mutations in the gene encoding succinate dehydrogenase (*SDH*), a mitochondrial complex II gene, lead to a decrease in its expression, suggesting its role in PCC development [31]. Interestingly, the expression of cytochrome c oxidase (*COX*) IV was extremely variable in mutant and wild-type PCC samples, suggesting mitochondrial disruption in these tumors, independent of genetic factors [31]. The mitochondrial open reading frame of 12S rRNA type-c (MOTS-c) is one of the newest mitochondrial-derived peptides (MDPs), first described in 2015, and has a wide range of physiological functions [33]. MDP proteins can be translocated to the nucleus under metabolic stress and act as nuclear receptors by promoting direct expression of nuclear genes to maintain cell homeostasis [34]. In addition, by secreting MOTS-c peptides into the bloodstream, they can act as hormone-like bioactive peptides that exert their effects on distant tissues throughout the body [33]. MOTS-c may be co-expressed and secreted by different tissues. MOTS-c may play an essential role in the endocrine system by regulating the muscle metabolism, insulin sensitivity and maintaining the cellular homeostasis [33,35,36,37,38,39,40,41]. Accordingly, the therapeutic strategies to increase MOTS-c level may have broad beneficial effects in the future.

Therefore, this study aimed to analyze samples of adrenal disease spectrum adrenocortical carcinoma, adrenocortical adenoma, and pheochromocytoma, and compare them to normal adrenal tissue. The objectives were to determine (*i*) whether the expression levels of the MOTS-c gene and protein vary among different types of adrenal tumors, and (*ii*) whether the localization of the MOTS-c protein is altered not only among the various adrenal tumors but also during the progression of ACC.

## 2. Results

### 2.1. MOTS-c mRNA Expression in Adrenal Disease Spectrum Samples—qRT-PCR

**Figure 1:**
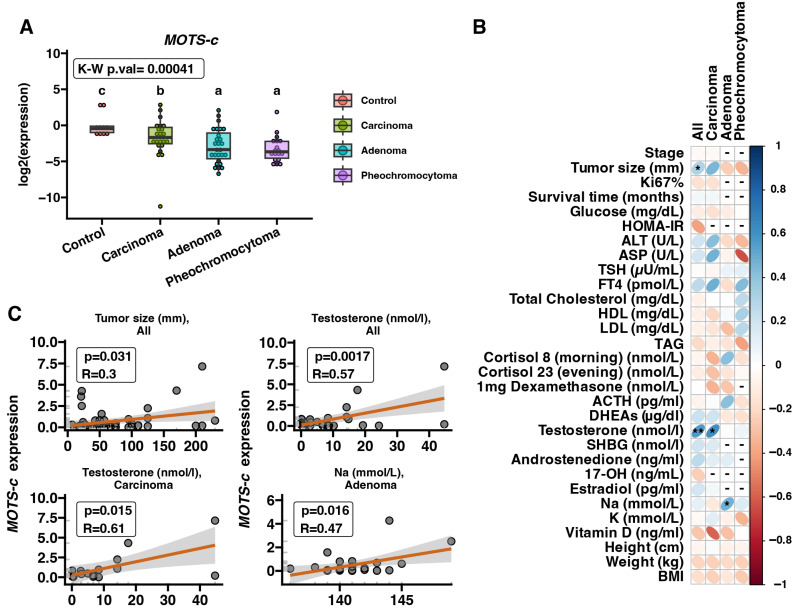
Adrenal expression of *MOTS-c* mRNA and correlations with demographic and laboratory features of patients with adrenal disease compared to heathy controls. *MOTS-c* gene expression is down-regulated in the spectrum of adrenal disease in comparison to healthy controls (normal adrenal tissue) (**A**). Correlations of MOTS-c mRNA expression with all clinical parameters of the patients (**B**), with the most significant and impactful correlations highlighted (**C**). Comparisons between the groups were performed using Kruskal–Wallis followed by the Dunn post hoc test. Data points are presented as dots on the corresponding boxplot. The differences between the groups are shown as letter annotations. Different letters indicate significant (*p* < 0.05) differences between the compared groups. Each boxplot indicates median and interquartile range (IQR) values. Correlation analysis was performed using Pearson correlation. Blue color indicates positive correlation while red indicates a negative correlation, intensity of the color reflects the strength of the correlation (R value). Dashes (“-”) signify missing patient data or when the number of patients with corresponding data is fewer than five in the respective group. *p*-value < 0.05 was considered statistically significant. * *p* < 0.05, ** *p* < 0.01.

The expression levels of the *MOTS-c* gene were analyzed in adrenal tissue samples obtained from patients diagnosed with ACC (*n* = 22), ACA (*n* = 28) and PCC (*n* = 18) as well as from healthy individuals (*n* = 10). Our results showed a statistically significant down-regulation (*p* = 0.00041) in *MOTS-c* gene expression in all cancer groups compared with controls. Interestingly, the *MOTS-c* gene expression was significantly decreased in ACA and PCC compared to both ACC and control (*p* < 0.05) (Figure 1A). Next, we performed a correlation analysis between *MOTS-c* gene expression in tumor samples and patients’ clinical data (Figure 1B). The results showed a positive correlation with tumor size (*p* = 0.031, R = 0.3) and testosterone levels (*p* = 0.0017, R = 0.57) in all analyzed groups. A positive correlation with testosterone levels (*p* = 0.015, R = 0.3) was also found in the ACC group and positively correlated with sodium levels (*p* = 0.016, R = 0.47) in the ACA group (Figure 1C). For other analyzed factors, we did not observe significant changes in the ACC, ACA, or PCC groups.

### 2.2. MOTS-c Protein Expression in Serum Samples—ELISA

**Figure 2:**
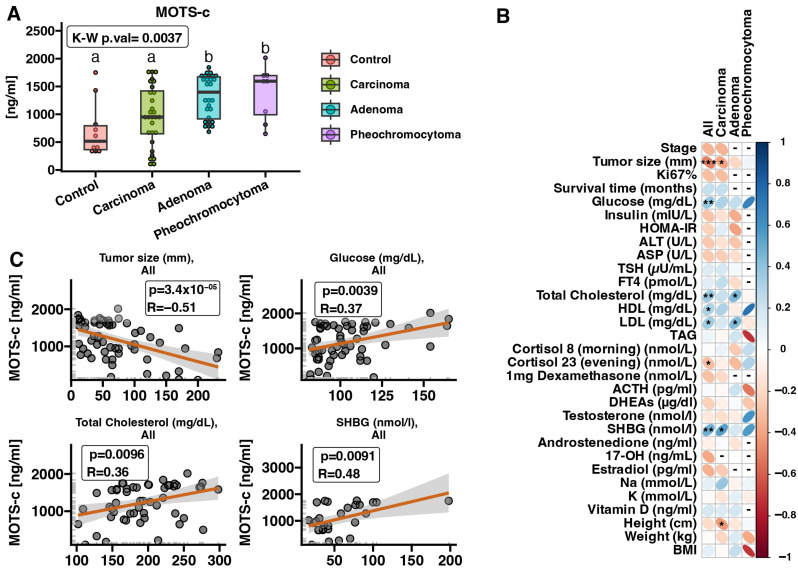
Serum expression of MOTS-c protein and correlations with demographic and laboratory features of patients with adrenal disease compared to heathy controls. MOTS-c protein expression is up-regulated in the spectrum of adrenal disease in comparison to healthy controls (normal adrenal tissue) (**A**). Correlations of MOTS-c protein expression with all clinical parameters of the patients (**B**), with the most significant and impactful correlations highlighted (**C**). Comparisons between the groups were performed using Kruskal–Wallis followed by Dunn post hoc test. All groups are presented as boxplots indicating median and interquartile range (IQR) values. Each data point is displayed as a dot on the corresponding boxplot. The differences between the groups are shown as letter annotations. Different letters indicate significant (*p* < 0.05) differences between the compared groups. Correlation analysis was performed using Pearson correlation. Blue color indicates positive correlation and red indicates negative correlation, while intensity of the color reflects the strength of the correlation (R value). Dashes (“-”) signify missing patient data or when the number of patients with corresponding data is fewer than five in the respective group. *p*-value < 0.05 was considered statistically significant. * *p* < 0.05, ** *p* < 0.01, *** *p* < 0.001.

Furthermore, we investigated the expression level of MOTS-c protein in serum samples of patients with adrenal tumors ACC (*n* = 29), ACA (*n* = 28), PCC (*n* = 8) and healthy controls (*n* = 10) using enzyme-linked immunosorbent assay (ELISA). The results revealed an increased expression of MOTS-c protein in serum samples from ACA and PCC patients compared to healthy individuals (*p* < 0.05) (Figure 2A). However, no significant difference was observed in MOTS-c protein levels in serum samples between patients with ACC and healthy individuals. Next, we correlated the results obtained from ELISA with the clinical characteristics of the patients. Correlation analysis of circulating MOTS-c protein expression and patient clinical data showed negative correlations with tumor size (*p* = 3.4 × 10^−5^, R = −0.51) and evening cortisol concentration among patients with adrenal tumors (Figure 2B). Moreover, we found that in all analyzed groups, the levels of glucose (*p* = 0.0039, R = 0.37), total cholesterol (*p* = 0.0096, R = 0.36), HDL, LDL, and sex hormone-binding globulin (SHBG) were positively correlated with MOTS-c protein expression (Figure 2B,C). In the context of the selected analyzed diseases, we revealed a positive correlation between MOTS-c protein expression and SHBG levels and negative correlations with tumor size and patient height in ACC patients. Moreover, in the serum of ACA patients we showed positive correlations between MOTS-c protein expression and total cholesterol, as well as LDL level.

### 2.3. MOTS-c Protein Expression in Adrenal Disease Spectrum—Tissue Microarray Slide

**Figure 3:**
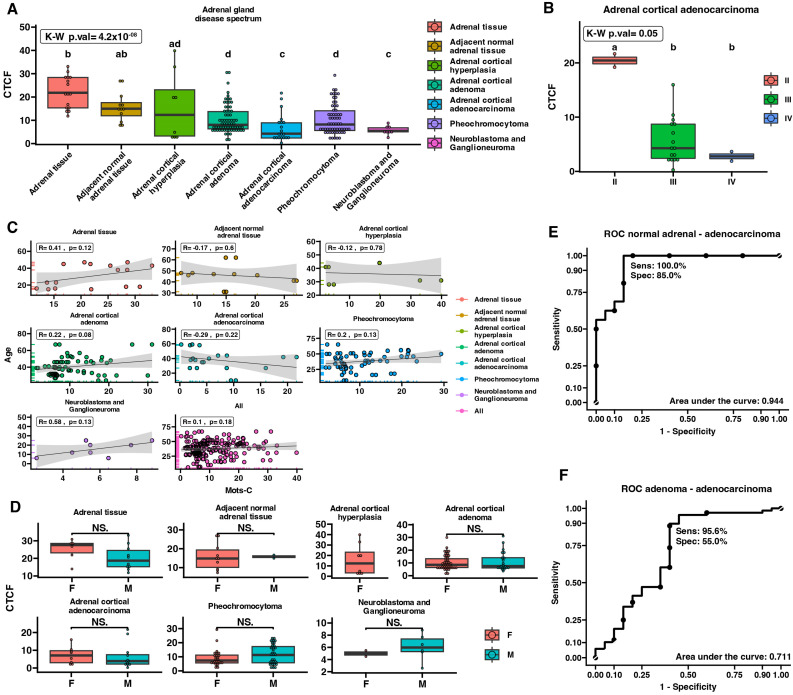
Adrenal expression of MOTS-c protein and correlations with demographic features of patients with adrenal disease compared to heathy controls. MOTS-c protein expression is down-regulated in the spectrum of adrenal disease in comparison to healthy controls (normal adrenal tissue) (**A**). MOTS-c protein expression is reduced within ACC progression (**B**). Correlations of MOTS-c protein expression with patient age (**C**) and gender (**D**) in all analyzed groups. The potential of MOTS-c protein as a diagnostic biomarker in distinguishing normal adrenal tissue from ACC (**E**), and ACA from ACC (**F**) was investigated. Comparisons between >2 groups were performed using Kruskal–Wallis followed by Dunn post hoc test. The differences between the groups are shown as letter annotations, where different letters indicate significant differences (*p* < 0.05) between the compared groups and presented as boxplots indicating median and interquartile range (IQR) values. Densitometric data from individual patients are displayed as dots on the corresponding graph. Correlation analysis was performed using Pearson correlation. Statistical analysis for comparison between 2 groups was performed using Mann–Whitney U test. The sensitivity, specificity, and area under curve (AUC) values are shown in both graphs of the ROC analysis.

**Figure 4:**
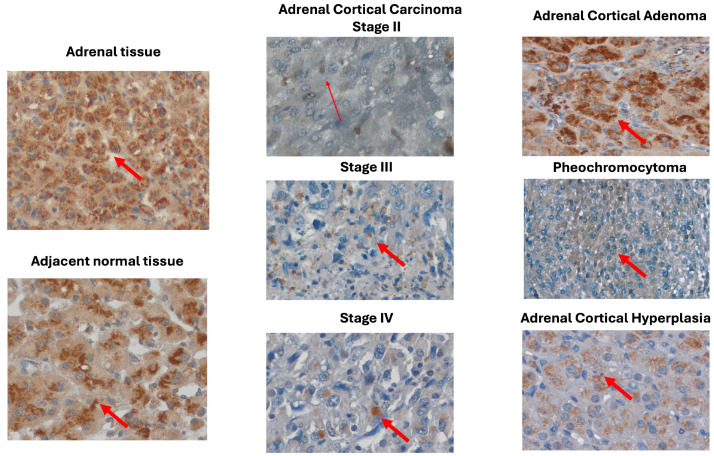
Representative images of MOTS-c protein immunohistochemical reactivity in the spectrum of adrenal disease on TMA slide, including stages II–IV ACC. Brown staining, pointed out by red arrows, indicates typical cytoplasmatic localization of MOTS-c protein with hematoxylin counterstaining to distinguish cell nuclei. Original magnifications 400×.

To analyze the expression and localization of MOTS-c peptide in various types of adrenal tumors, we performed densitometric analysis of a tissue microarray (TMA) slide containing adrenal disease spectrum samples. Using densitometric analysis, we showed that the protein expression of MOTS-c was diminished in all analyzed groups of malignant adrenal tissue in comparison with normal adrenal tissue, as well as in comparison with adjacent normal tissue (Figure 3A). Moreover, we evaluated differences in MOTS-c protein levels during ACC progression (Figure 3B). The analysis revealed that the level of MOTS-c peptide expression gradually decreased in further stages of ACC (*p* = 0.05), especially in stages III and IV of the disease, compared with stage II, but the difference was not statistically significant. Moreover, we performed a correlation analysis between MOTS-c protein expression in the TMA slide and the available patient characteristic. We indicate that the expression of MOTS-c peptide was not correlated with patient age (Figure 3C) or sex (Figure 3D) across all the analyzed groups. We also investigated the potential of tissue MOTS-c protein expression as a diagnostic biomarker for differentiating adrenal tumor types. The ROC curve analysis showed that MOTS-c might be used to distinguish ACC samples (sensitivity = 100%, specificity = 85.0%, AUC = 0.944) from normal adrenal tissue (Figure 3E), but not ACA from ACC due to low specify (sensitivity = 95.6%, specificity = 55.0%, AUC = 0.711) (Figure 3F). Furthermore, immunohistochemical staining was performed to determine the protein localization of MOTS-c in adrenal tissue samples (Figure 4). We found that the MOTS-c protein had predominantly cytoplasmic localization in all analyzed samples. Furthermore, microscopic analysis confirmed the densitometric results, especially the decreasing amount of protein during ACC progression. We did not detect MOTS-c protein in the cell nuclei, regardless of tumor type.

## 3. Discussion

Adrenal cancers such as ACC, ACA, and PCC are complex heterogeneous diseases with poorly understood etiologies and limited treatment options. Recent research has underscored the crucial role of mitochondria and mitochondrial-derived peptides in the survival, proliferation, and metabolic adaptation of cancer cells. In this study, we conducted a comprehensive analysis of *MOTS-c* mRNA and protein expression in tissue and serum samples from patients with three different types of adrenal tumors and compared them with healthy controls. Our findings revealed a general decrease in *MOTS-c* mRNA expression across all adrenal tumors analyzed. Moreover, we showed that these patients were characterized by increased expression of MOTS-c protein in the serum, especially in the ACA and PCC groups, but not in the ACC. Furthermore, we showed that the local distribution of MOTS-c protein in adrenal tissue from TMA slides decreased in all analyzed adrenal tumors. Interestingly, we showed that the expression of MOTS-c protein is down-regulated during ACC progression (stages III and IV), but does not correlate with patient age and sex. The correlation of the results with clinical data indicated that tumor size and testosterone levels were positively correlated with *MOTS-c* mRNA and negatively correlated with serum MOTS-c protein expression. In addition, serum MOTS-c protein was positively correlated with glucose, total cholesterol, HDL, LDL, and SHGB levels in all analyzed groups compared to the control.

To our knowledge, this is the first study to describe a detailed examination of *MOTS-c* mRNA and protein expression in ACC, ACA, and PCC. The functions of MOTS-c are still being explored, and only a limited number of studies have investigated the expression of MOTS-c peptide in cancer cases, but not in adrenal tumors [42,43,44]. Furthermore, the TCGA database does not provide any information on MOTS-c expression in various cancer types [45]. Here, we sought to identify changes in the expression profile of *MOTS-c* mRNA and protein in adrenal tissues of patients with ACC, ACA, and PCC. Our study revealed that *MOTS-c* mRNA expression was inhibited in all adrenal tumors analyzed. Furthermore, we confirmed the disturbed local distribution of MOTS-c peptide in the adrenal glands, especially under ACC conditions, due to its reduced expression during disease progression. Moreover, we did not find any correlation between MOTS-c protein expression and the age or sex of adrenal samples from patients with different adrenal disorders.

Studies on prostate cancer by Ramirez-Torrez et al. [42] indicated that enhanced serum MOTS-c levels are associated with a lower risk of disease development and progression. Other studies investigating the expression of MOTS-c protein in the serum of patients with breast cancer did not indicate any significant changes compared with controls, even after metformin administration [43]. The authors explained that metformin and MOTS-c, whose main effects are concentrated in the muscles, probably have different targets. Another study analyzed the serum distribution of MOTS-c in breast cancer survivors after 16 weeks of aerobic and resistance exercise interventions [44]. They found that the level of MOTS-c in the serum significantly increased after exercise. Moreover, these results correlated with body weight, fat mass, HOMA-IR, CRP reduction, and improvement in lean mass [44]. Nevertheless, a major source of circulating MOTS-c is muscles, which has attracted growing interest in research on this phenomenon and the role that MOTS-c plays in the human aging process [41,46]. It is well known that MOTS-c expression and concentration in serum decreases with age and contributes to aging and age-related diseases [41,46,47]. Our analysis indicated that the serum expression of MOTS-c protein was significantly up-regulated in ACA and PCC but not in ACC, where we observed a non-significant increase in expression. Evidence has confirmed that the adrenal glands are not the main source of MOTS-c secretion. Moreover, the increased secretion of MOTS-c into the serum from other sources may be the result of an adaptive mechanism, whereby an insufficient amount of MOTS-c at the site of disease is compensated by systemic synthesis. Our findings seem to demonstrate that reduced expression of MOTS-c at both the mRNA and protein levels suggests disrupted or inhibited local distribution in the adrenal glands of patients with adrenal tumors. First, disease progression not only requires architectural reorganization of the tissue but also reduces the number of properly functioning cells. Second, although there was a reduction in the number of functional mitochondria, it might have led to disruptions in the transcription and distribution of *MOTS-c*. In addition, the concomitant increase in mitochondrial dysfunction disrupts cellular homeostasis, which is normally maintained by mitochondria.

Cholesterol is a precursor for the synthesis of steroid hormones during steroidogenesis, in which the mitochondria play an essential role [48,49]. Our study identified positive correlations between serum MOTS-c protein levels and testosterone, total cholesterol, and sex hormone-binding globulin (SHBG). These findings suggest that MOTS-c may play a constructive role in facilitating the release and transport of testosterone into the bloodstream. We also observed an adverse correlation between MOTS-c protein expression in serum samples and evening cortisol levels in patients with adrenal tumors. Adrenal tumors are often associated with dysregulated hormone secretion and aldosteronism, which can lead to various metabolic disorders. Several studies have linked adrenal tumors and aldosteronism to cardiovascular disease and diabetes [50,51,52], possibly linking the metabolic changes associated with adrenal tumors to the protective effects of MOTS-c peptide against these changes. Furthermore, MOTS-c is known for its function in maintaining glucose homeostasis by protecting against insulin resistance and hyperglycemia [33,53,54,55]. However, in contrast to these findings, our results showed a positive correlation between circulating MOTS-c protein levels and blood glucose concentration. This result suggests that under certain conditions, due to the focus on fighting cancer, the amount of MOTS-c may not be sufficient to fight glucose disorders.

The opposing expression patterns of MOTS-c in serum and tissues suggest post-translational modifications of the peptide in tumor cells. These modifications, associated with malignant cell transformation, may lead to increased degradation of the MOTS-c protein and its reduced expression in cancer cells. Additionally, changes in the peptide export process and mitochondrial dysfunction related to metabolic stress in patients with cancer may also influence the observed differences in MOTS-c expression.

Our study had several limitations that need to be acknowledged. First, the relatively small sample size and sample collection method did not allow us to perform more advanced analyses, such as western blotting. Second, the lack of paired serum and tissue samples from the same patient limited our ability to draw direct correlations between these compartments, potentially affecting the interpretation of our findings. Third, potential confounding factors, such as variations in patient treatment and lifestyle differences, were not fully controlled. These factors could influence the results and should be addressed in future research.

## 4. Materials and Methods

### 4.1. Patients’ Characteristics

The study included 67 tumor tissue samples from patients who underwent adrenalectomy for ACC (*n* = 22), ACA (*n* = 27), and PCC (*n* = 18). Clinical data were collected from the patients prior to adrenalectomy and tissue sampling. For molecular analysis, pathologically altered adrenal specimens (≈0.5 cm^3^) were collected and stored in RNAlater™ (#R0901, Sigma, St. Louis, MO, USA) for subsequent analysis of mRNA expression. Unchanged adrenal samples from kidney donors were used as the controls (*n* = 10). In addition, serum was collected from 65 patients with adrenal tumors, including ACC (*n* = 29), ACA (*n* = 28), and PCC (*n* = 8) patients, and 10 samples from healthy blood donors as controls. Serum was collected into the serum separator tubes, then the samples were allowed to clot for 2 h at room temperature at 4 °C before centrifugation at 1000× *g* for 20 min. Subsequently, the serum was aliquoted and stored at −20 °C for ELISA. The research protocol was approved by the Local Ethics Committee of Poznan University of Medical Sciences (decision no. 31/22) and adhered to the tenets of the Declaration of Helsinki. Clinical characteristics were gathered during the initial diagnostic visits through standardized interviews and evaluations conducted by two physicians. The characteristics of the patients included in both qPCR and ELISA are shown in Table 1 and Table 2, respectively.

### 4.2. RNA Extraction and Quantification of the Gene Expression

qPCR was performed in accordance with MIQE guidelines [56]. Total RNA was extracted from adrenal gland tissue using RL reagent from the Universal RNA Purification Kit (#E3599-02, EURx, Gdańsk, Poland) with the addition of β-mercaptoethanol to inactivate RNase. RNA isolation and purification were conducted according to the instructions provided with the Universal RNA Purification Kit (#E3599-02, EURx, Poland). The quantity of total mRNA was determined by measuring the optical density at 260 nm, and its purity was assessed by calculating the 260/280 nm absorption ratio (greater than 1.8) using a NanoDrop spectrophotometer (Thermo Fisher, Waltham, MA, USA). cDNA synthesis was conducted using the TaqManTM MicroRNA Reverse Transcription Kit (#4366597, ThermoFisher, Carlsbad, CA, USA) and TaqMan™ microRNA assays with custom primers for MOTS-c (#4398988, ThermoFisher, Carlsbad, CA, USA) and predesigned primers for the reference gene RNU48 (#4427975, ThermoFisher, Carlsbad, CA, USA) [41]. In total, 500 μg/mL of RNA was used for each sample. The synthesized cDNA was stored at −20 °C. The expression of target genes was analyzed according to the manufacturer’s protocol by quantitative real-time PCR using TaqMan Fast Advanced Master Mix (#4444557, ThermoFisher, Carlsbad, CA, USA). Gene expression was quantified using the CFX96 Touch Real-Time PCR Detection System (CFX96, Bio-Rad, Hercules, CA, USA) in a 20 μL reaction mixture according to the manufacturer’s instructions. All samples were amplified in duplicates. The relative expression of target genes was calculated using the ΔΔCt quantification method.

### 4.3. Quantifying MOTS-c Protein Levels Using ELISA

MOTS-c protein levels in the serum of patients with adrenal tumors and controls were quantified using an ELISA kit for human MOTS-c (#CEX132Hu, Cloud-Clone Corp., Houston, TX, USA), in accordance with the manufacturer’s instructions. The absorbance was determined by measuring the optical density at 450 nm (Biotek, Winooski, VT, USA, Synergy 2). A quantitative analysis was conducted using a four-parameter logistic curve (4PL) from the “drc” Bioconductor package [57].

### 4.4. Immunohistochemical Analyses

An unstained adrenal gland disease spectrum (adrenal cancer progression) TMA slide (AD2081, US Biomax, Inc., Rockville, MD, USA) was used. The slide contained 34 cases of ACAs, 10 ACCs, 3 neuroblastomas, 1 ganglioneuroma, 30 PCCs, 4 hyperplasias, and 6 adjacent normal tissue samples. Each case was represented by duplicate cores on a slide. IHC analyses were performed as in the previously described procedures [58,59,60]. Initially, TMA sections were deparaffinized, briefly rehydrated in decreasing concentrations of ethanol, and rinsed with phosphate-buffered saline (PBS). Antigen unmasking was performed using citrate-based, pH 6.1 Antigen Unmasking Solution (#H-3300-250, Vector). The slides were washed and incubated with Normal Horse Serum (2.5%) for 20 min. Subsequently, the sections were incubated with anti-MOTS-c antibody (MOTSC-101AP, FabGennix, Frisco, TX, USA) at a concentration of 1:300 overnight at 4 °C. After incubation, the samples were stained using an ImmPRESS^®^ HRP Universal PLUS Polymer Kit (MP-7800, Vector Laboratories, Inc., Newark, CA, USA). The sections were then counterstained with Mayer’s hematoxylin (#S330930-2, DAKO, Agilent, Santa Clara, CA, USA), dehydrated, and mounted. The entire TMA slide was digitized using a GRUNDIUM OCUS^®^20 slide scanner (Grundium, Tampere, Finland). IHC staining was analyzed and documented at a high magnification using CaseViewer 2.3 (64-bit version) for Windows (3D Histech Ltd., Budapest, Hungary).

### 4.5. Semiquantitative Evaluation of MOTS-c Protein Expression

The expression of MOTS-c protein on the TMA slide was quantified using the densitometric method. First, the blue-violet color obtained by hematoxylin staining was removed from the scanned slide using Adobe Photoshop ver. 21.1.0 (Adobe Inc., San Jose, CA, USA). The resulting image with remaining brown staining indicative of the specific IHC reaction was saved in TIFF format and loaded into the ImageJ software (version 1.5q, Wayne Rasband, National Institutes of Health, Bethesda, MD, USA). Subsequently, densitometric analysis was conducted in accordance with the Open Lab Book protocol, adapted to the TMA format [61]. The integrated density was calculated for each TMA sample, with a fixed diameter covering of 8800 pixels per piece. The background signal was quantified and incorporated into the final calculation of the measured pixel intensities for each tissue array core. The densitometric values obtained are presented as boxplots, with the median and interquartile range (IQR) displayed. Densitometric data from individual patients are displayed as dots overlaid on the corresponding box plots. Furthermore, to assess the potential of MOTS-c as a biomarker for differentiating between different types of adrenal tumors and healthy tissue, we generated a receiver operating characteristic (ROC) curve plot using the ‘pROC’ library [62].

### 4.6. Statistical Analysis

Statistical analyses were conducted using the R programming language (version 4.1.2; R Core Team 2021), supported by the ‘ggplot2’ [63] and ‘ggprism’ [63] libraries for the visualization of the results. Comparisons between two groups were evaluated using the Mann–Whitney U test. For analyses involving more than two groups, the Kruskal–Wallis test was applied, followed by Dunn’s post hoc test. Group differences were indicated by letters, with different letters indicating statistically significant differences (*p* < 0.05). Correlation analyses were conducted between patient characteristics and MOTS-c expression, employing Pearson correlation, with a significance level of *p* < 0.05. Correlation matrices were constructed using the corrplot package for R [64].

## 5. Conclusions

To our knowledge, this is the first study to investigate MOTS-c expression in patients with adrenal tumors, particularly ACC, ACA, and PCC. The results of this study indicate that (*i*) *MOTS-c* mRNA expression is down-regulated in all analyzed adrenal tumors, whereas serum MOTS-c protein levels are increased in the ACA and PCC. Moreover, (*ii*) the tissue distribution of MOTS-c peptide was diminished in adrenal tumor samples, and this reduction was also observed during ACC progression. There are several possible explanations for this observation. First, mitochondrial homeostasis may be disrupted by increased mitochondrial dysfunction through a decrease in the number of fully functional organelles. Second, disease progression requires changes in the tissue structure by reducing the number of healthy adrenal cells. Furthermore, correlations with clinical outcomes might provide new insights into the potential role of MOTS-c in regulating hormonal activity of the endocrine system. MOTS-c plays an important role in cancer and has the potential to be used as a therapeutic target. However, the exact effects of MOTS-c on carcinogenesis, tumor growth, and metastasis, and the mechanism of differential expression of MOTS-c in tumor tissues and circulation, are not fully understood and require further research.

## Figures and Tables

**Table 1:** Clinical characteristics of patients with adrenocortical carcinoma (ACC), adrenocortical adenoma (ACA), and pheochromocytoma (PCC) included in qPCR analysis.

Characteristic Mean (min–max)	ACC	ACA	PCC
Sex (male/female)	9/13	4/23	9/9
Age (years)	54 (27–82)	62.3 (30–86)	58.1 (31–82)
Tumor size (mm)	130.5 (57–230)	39.1 (7–67)	44.7 (19–75)
Glucose (mg/dL)	136.9 (83–466)	102.3 (73–175)	118.8 (81–185)
Total cholesterol (mg/dL)	154.4 (109–203)	198.8 (146–266)	217.4 (130–298)
HDL (mg/dL)	42.8 (31–62)	67.2 (39–119)	68.9 (37–121)
Testosterone (nmol/L)	10.3 (0.2–44.7)	4.8 (0.1–24.3)	8.0 (0.8–19.3)
NA (mmol/L)	140.5 (137–145)	141.3 (136–149)	141.1 (138–144)
K (mmol/L)	4.4 (3.1–5.3)	4.2 (2.4–5.2)	4.6 (4–5.4)
Height (cm)	167.1 (156–180)	163.4 (149–175)	171 (158–188)
Weight (kg)	71.7 (56–89)	74.2 (52–104)	73.4 (51–107)
BMI	25.7 (17.9–31.2)	27.1 (17.9–37.6)	24.4 (18.8–31.1)

**Table 2:** Clinical characteristics of patients with adrenocortical carcinoma (ACC), adrenocortical adenoma (ACA), and pheochromocytoma (PCC) included in ELISA analysis.

Characteristic Mean (min–max)	ACC	ACA	PCC
Sex (male/female)	9/20	8/20	8/0
Age (years)	53 (25–74)	61 (27–81)	45 (33–65)
Tumor size (mm)	116.6 (45–232)	28.3 (11–53)	57.9 (37–75)
Glucose (mg/dL)	98.6 (81–154)	103.8 (85–165)	120.1 (98–154)
Total cholesterol (mg/dL)	180.3 (102–277)	190.5 (131–274)	240.5 (170–298)
HDL (mg/dL)	50.4 (21–82)	57.7 (32–79)	61.2 (48–85)
Testosterone (nmol/L)	9.1 (0.2–52)	4.5 (0.1–19.4)	18.7 (10.2–26.6)
Na (mmol/L)	141.7 (137–146)	141.7 (136–150)	140 (135–143)
K (mmol/L)	4.5 (3.3–5.6)	4.5 (3.8–5.3)	4.7 (4.3–5)
Height (cm)	167.4 (156–183)	167.24 (146–198)	175.7 (162–196)
Weight (kg)	76.8 (54–133)	82 (55–150)	68.5 (54–85)
BMI	27.4 (17.9–41)	29.4 (20.5–55)	22.1 (19–24.6)

## Data Availability

All of the data discussed in this work, if not already included in the manuscript, are available from the corresponding author on reasonable request.

## References

[1] Mansmann G., Lau J., Balk E., Rothberg M., Miyachi Y., Bornstein S.R. (2004). The clinically inapparent adrenal mass: Update in diagnosis and management. Endocr. Rev..

[2] Corssmit E.P.M., Dekkers O.M. (2019). Screening in adrenal tumors. Curr. Opin. Oncol..

[3] Fassnacht M., Arlt W., Bancos I., Dralle H., Newell-Price J., Sahdev A., Tabarin A., Terzolo M., Tsagarakis S., Dekkers O.M. (2016). Management of adrenal incidentalomas: European Society of Endocrinology Clinical Practice Guideline in collaboration with the European Network for the Study of Adrenal Tumors. Eur. J. Endocrinol..

[4] Else T., Kim A.C., Sabolch A., Raymond V.M., Kandathil A., Caoili E.M., Jolly S., Miller B.S., Giordano T.J., Hammer G.D. (2014). Adrenocortical carcinoma. Endocr. Rev..

[5] Bilimoria K.Y., Shen W.T., Elaraj D., Bentrem D.J., Winchester D.J., Kebebew E., Sturgeon C. (2008). Adrenocortical carcinoma in the United States: Treatment utilization and prognostic factors. Cancer.

[6] Bechmann N., Moskopp M.L., Constantinescu G., Stell A., Ernst A., Berthold F., Westermann F., Jiang J., Lui L., Nowak E. (2024). Asymmetric Adrenals: Sexual Dimorphism of Adrenal Tumors. J. Clin. Endocrinol. Metab..

[7] Audenet F., Mejean A., Chartier-Kastler E., Roupret M. (2013). Adrenal tumours are more predominant in females regardless of their histological subtype: A review. World J. Urol..

[8] Allolio B., Fassnacht M. (2006). Clinical review: Adrenocortical carcinoma: Clinical update. J. Clin. Endocrinol. Metab..

[9] Fassnacht M., Allolio B. (2009). Clinical management of adrenocortical carcinoma. Best. Pract. Res. Clin. Endocrinol. Metab..

[10] Luton J.P., Cerdas S., Billaud L., Thomas G., Guilhaume B., Bertagna X., Laudat M.H., Louvel A., Chapuis Y., Blondeau P. (1990). Clinical features of adrenocortical carcinoma, prognostic factors, and the effect of mitotane therapy. N. Engl. J. Med..

[11] Giordano T.J., Thomas D.G., Kuick R., Lizyness M., Misek D.E., Smith A.L., Sanders D., Aljundi R.T., Gauger P.G., Thompson N.W. (2003). Distinct transcriptional profiles of adrenocortical tumors uncovered by DNA microarray analysis. Am. J. Pathol..

[12] Assie G., Letouze E., Fassnacht M., Jouinot A., Luscap W., Barreau O., Omeiri H., Rodriguez S., Perlemoine K., Rene-Corail F. (2014). Integrated genomic characterization of adrenocortical carcinoma. Nat. Genet..

[13] Tissier F., Cavard C., Groussin L., Perlemoine K., Fumey G., Hagnere A.M., Rene-Corail F., Jullian E., Gicquel C., Bertagna X. (2005). Mutations of beta-catenin in adrenocortical tumors: Activation of the Wnt signaling pathway is a frequent event in both benign and malignant adrenocortical tumors. Cancer Res..

[14] Pinto E.M., Chen X., Easton J., Finkelstein D., Liu Z., Pounds S., Rodriguez-Galindo C., Lund T.C., Mardis E.R., Wilson R.K. (2015). Genomic landscape of paediatric adrenocortical tumours. Nat. Commun..

[15] Gara S.K., Lack J., Zhang L., Harris E., Cam M., Kebebew E. (2018). Metastatic adrenocortical carcinoma displays higher mutation rate and tumor heterogeneity than primary tumors. Nat. Commun..

[16] Ebbehoj A., Li D., Kaur R.J., Zhang C., Singh S., Li T., Atkinson E., Achenbach S., Khosla S., Arlt W. (2020). Epidemiology of adrenal tumours in Olmsted County, Minnesota, USA: A population-based cohort study. Lancet Diabetes Endocrinol..

[17] Parikh P.P., Rubio G.A., Farra J.C., Lew J.I. (2017). Nationwide review of hormonally active adrenal tumors highlights high morbidity in pheochromocytoma. J. Surg. Res..

[18] Li D., El Kawkgi O.M., Henriquez A.F., Bancos I. (2020). Cardiovascular risk and mortality in patients with active and treated hypercortisolism. Gland. Surg..

[19] Amar L., Bertherat J., Baudin E., Ajzenberg C., Bressac-de Paillerets B., Chabre O., Chamontin B., Delemer B., Giraud S., Murat A. (2005). Genetic testing in pheochromocytoma or functional paraganglioma. J. Clin. Oncol..

[20] Goldstein R.E., O’Neill J.A., Holcomb G.W., Morgan W.M., Neblett W.W., Oates J.A., Brown N., Nadeau J., Smith B., Page D.L. (1999). Clinical experience over 48 years with pheochromocytoma. Ann. Surg..

[21] Farrugia F.A., Charalampopoulos A. (2019). Pheochromocytoma. Endocr. Regul..

[22] Bryant J., Farmer J., Kessler L.J., Townsend R.R., Nathanson K.L. (2003). Pheochromocytoma: The expanding genetic differential diagnosis. J. Natl. Cancer Inst..

[23] Neumann H.P., Bausch B., McWhinney S.R., Bender B.U., Gimm O., Franke G., Schipper J., Klisch J., Altehoefer C., Zerres K. (2002). Germ-line mutations in nonsyndromic pheochromocytoma. N. Engl. J. Med..

[24] Liu Y., Sun Y., Guo Y., Shi X., Chen X., Feng W., Wu L.L., Zhang J., Yu S., Wang Y. (2023). An Overview: The Diversified Role of Mitochondria in Cancer Metabolism. Int. J. Biol. Sci..

[25] Zong Y., Li H., Liao P., Chen L., Pan Y., Zheng Y., Zhang C., Liu D., Zheng M., Gao J. (2024). Mitochondrial dysfunction: Mechanisms and advances in therapy. Signal Transduct. Target. Ther..

[26] Midzak A., Papadopoulos V. (2016). Adrenal Mitochondria and Steroidogenesis: From Individual Proteins to Functional Protein Assemblies. Front. Endocrinol..

[27] Nunnari J., Suomalainen A. (2012). Mitochondria: In sickness and in health. Cell.

[28] Gorman G.S., Chinnery P.F., DiMauro S., Hirano M., Koga Y., McFarland R., Suomalainen A., Thorburn D.R., Zeviani M., Turnbull D.M. (2016). Mitochondrial diseases. Nat. Rev. Dis. Primers.

[29] Wang J., Zhou H. (2020). Mitochondrial quality control mechanisms as molecular targets in cardiac ischemia-reperfusion injury. Acta Pharm. Sin. B.

[30] Bornstein S.R., Brown J.W., Carballeira A., Goodman J., Scherbaum W.A., Fishman L.M. (1996). Ultrastructural dynamics of mitochondrial morphology in varying functional forms of human adrenal cortical adenoma. Horm. Metab. Res..

[31] Rapizzi E., Ercolino T., Canu L., Giache V., Francalanci M., Pratesi C., Valeri A., Mannelli M. (2012). Mitochondrial function and content in pheochromocytoma/paraganglioma of succinate dehydrogenase mutation carriers. Endocr. Relat. Cancer.

[32] Chuang C.H., Dorsch M., Dujardin P., Silas S., Ueffing K., Holken J.M., Yang D., Winslow M.M., Gruner B.M. (2021). Altered Mitochondria Functionality Defines a Metastatic Cell State in Lung Cancer and Creates an Exploitable Vulnerability. Cancer Res..

[33] Lee C., Zeng J., Drew B.G., Sallam T., Martin-Montalvo A., Wan J., Kim S.J., Mehta H., Hevener A.L., de Cabo R. (2015). The mitochondrial-derived peptide MOTS-c promotes metabolic homeostasis and reduces obesity and insulin resistance. Cell Metab..

[34] Kim K.H., Son J.M., Benayoun B.A., Lee C. (2018). The Mitochondrial-Encoded Peptide MOTS-c Translocates to the Nucleus to Regulate Nuclear Gene Expression in Response to Metabolic Stress. Cell Metab..

[35] Kong B.S., Min S.H., Lee C., Cho Y.M. (2021). Mitochondrial-encoded MOTS-c prevents pancreatic islet destruction in autoimmune diabetes. Cell Rep..

[36] Lee C., Kim K.H., Cohen P. (2016). MOTS-c: A novel mitochondrial-derived peptide regulating muscle and fat metabolism. Free Radic. Biol. Med..

[37] Du C., Zhang C., Wu W., Liang Y., Wang A., Wu S., Zhao Y., Hou L., Ning Q., Luo X. (2018). Circulating MOTS-c levels are decreased in obese male children and adolescents and associated with insulin resistance. Pediatr. Diabetes.

[38] Kang G.M., Min S.H., Lee C.H., Kim J.Y., Lim H.S., Choi M.J., Jung S.B., Park J.W., Kim S., Park C.B. (2021). Mitohormesis in Hypothalamic POMC Neurons Mediates Regular Exercise-Induced High-Turnover Metabolism. Cell Metab..

[39] Xiao J., Zhang Q., Shan Y., Ye F., Zhang X., Cheng J., Wang X., Zhao Y., Dan G., Chen M. (2023). The Mitochondrial-Derived Peptide (MOTS-c) Interacted with Nrf2 to Defend the Antioxidant System to Protect Dopaminergic Neurons Against Rotenone Exposure. Mol. Neurobiol..

[40] Lu H., Wei M., Zhai Y., Li Q., Ye Z., Wang L., Luo W., Chen J., Lu Z. (2019). MOTS-c peptide regulates adipose homeostasis to prevent ovariectomy-induced metabolic dysfunction. J. Mol. Med..

[41] D’Souza R.F., Woodhead J.S.T., Hedges C.P., Zeng N., Wan J., Kumagai H., Lee C., Cohen P., Cameron-Smith D., Mitchell C.J. (2020). Increased expression of the mitochondrial derived peptide, MOTS-c, in skeletal muscle of healthy aging men is associated with myofiber composition. Aging.

[42] Ramirez-Torres A., Reagan A.L., Howard L.E., Wiggins E., Vidal A.C., Wan J., Miller B., Freedland S.J., Cohen P. (2022). Racial differences in circulating mitochondria-derived peptides may contribute to prostate cancer health disparities. Prostate.

[43] Cuyas E., Verdura S., Martin-Castillo B., Menendez J.A., METTEN Study Group (2022). Circulating levels of MOTS-c in patients with breast cancer treated with metformin. Aging.

[44] Dieli-Conwright C.M., Sami N., Norris M.K., Wan J., Kumagai H., Kim S.J., Cohen P. (2021). Effect of aerobic and resistance exercise on the mitochondrial peptide MOTS-c in Hispanic and Non-Hispanic White breast cancer survivors. Sci. Rep..

[45] The Cancer Genome Atlas. https://www.cancer.gov/ccg/research/genome-sequencing/tcga.

[46] Reynolds J.C., Lai R.W., Woodhead J.S.T., Joly J.H., Mitchell C.J., Cameron-Smith D., Lu R., Cohen P., Graham N.A., Benayoun B.A. (2021). MOTS-c is an exercise-induced mitochondrial-encoded regulator of age-dependent physical decline and muscle homeostasis. Nat. Commun..

[47] Kim S.J., Miller B., Kumagai H., Silverstein A.R., Flores M., Yen K. (2021). Mitochondrial-derived peptides in aging and age-related diseases. Geroscience.

[48] Gomez-Sanchez C.E., Gomez-Sanchez E.P. (2024). Cholesterol Availability and Adrenal Steroidogenesis. Endocrinology.

[49] Miller W.L. (2011). Role of mitochondria in steroidogenesis. Endocr. Dev..

[50] Quinkler M., Born-Frontsberg E., Fourkiotis V.G. (2010). Comorbidities in primary aldosteronism. Horm. Metab. Res..

[51] Hanslik G., Wallaschofski H., Dietz A., Riester A., Reincke M., Allolio B., Lang K., Quack I., Rump L.C., Willenberg H.S. (2015). Increased prevalence of diabetes mellitus and the metabolic syndrome in patients with primary aldosteronism of the German Conn’s Registry. Eur. J. Endocrinol..

[52] La Batide-Alanore A., Chatellier G., Plouin P.F. (2003). Diabetes as a marker of pheochromocytoma in hypertensive patients. J. Hypertens..

[53] Yin Y., Pan Y., He J., Zhong H., Wu Y., Ji C., Liu L., Cui X. (2022). The mitochondrial-derived peptide MOTS-c relieves hyperglycemia and insulin resistance in gestational diabetes mellitus. Pharmacol. Res..

[54] Kirik A., Dogru T., Yanik B., Sen H., Eroglu M., Baykan O., Bozyel E.A., Ergene A., Selcuk E., Tasci I. (2023). The relationship of circulating MOTS-c level with liver fibrosis and metabolic components in patients with metabolic dysfunction-associated fatty liver disease. Eur. Rev. Med. Pharmacol. Sci..

[55] Ramanjaneya M., Bettahi I., Jerobin J., Chandra P., Abi Khalil C., Skarulis M., Atkin S.L., Abou-Samra A.B. (2019). Mitochondrial-Derived Peptides Are Down Regulated in Diabetes Subjects. Front. Endocrinol..

[56] Bustin S.A., Benes V., Garson J.A., Hellemans J., Huggett J., Kubista M., Mueller R., Nolan T., Pfaffl M.W., Shipley G.L. (2009). The MIQE guidelines: Minimum information for publication of quantitative real-time PCR experiments. Clin. Chem..

[57] Ritz C., Baty F., Streibig J.C., Gerhard D. (2015). Dose-Response Analysis Using R. PLoS ONE.

[58] Blatkiewicz M., Kaminski K., Szyszka M., Al-Shakarchi Z., Olechnowicz A., Stelcer E., Komarowska H., Tyczewska M., Klimont A., Karczewski M. (2023). The Enhanced Expression of ZWILCH Predicts Poor Survival of Adrenocortical Carcinoma Patients. Biomedicines.

[59] Komarowska H., Malinska A., Komekbai Z., Brominska B., Bednarek-Rajewska K., Ruchala M., Rucinski M. (2021). Immunohistochemical analysis of ghrelin expression in various types of adrenal tumors. Folia Histochem. Cytobiol..

[60] Blatkiewicz M., Szyszka M., Olechnowicz A., Kaminski K., Jopek K., Komarowska H., Tyczewska M., Klimont A., Wierzbicki T., Karczewski M. (2024). Impaired Expression of Humanin during Adrenocortical Carcinoma. Int. J. Mol. Sci..

[61] The Open Lab Book. https://theolb.readthedocs.io/en/latest/imaging/measuring-cell-fluorescence-using-imagej.html.

[62] Robin X., Turck N., Hainard A., Tiberti N., Lisacek F., Sanchez J.C., Muller M. (2011). pROC: An open-source package for R and S+ to analyze and compare ROC curves. BMC Bioinform..

[63] ggprism: A ‘ggplot2’ Extension Inspired by ‘GraphPad Prism’. https://csdaw.github.io/ggprism/authors.html#citation.

[64] An Introduction to corrplot Package. https://cran.r-project.org/web/packages/corrplot/vignettes/corrplot-intro.html.

